# Short-Term Effects of Heart Rate Variability Biofeedback on Working Memory

**DOI:** 10.1007/s10484-024-09624-7

**Published:** 2024-02-16

**Authors:** Mariam Bahameish, Tony Stockman

**Affiliations:** 1 College of Science and Engineering, Hamad Bin Khalifa University, Doha, Qatar; 2 School of Electronics Engineering and Computer Science, Queen Mary University of London, London, UK

**Keywords:** Heart rate variability, Biofeedback, Vagal tone, Working memory, Cognitive performance

## Abstract

Drawing upon the well-documented impact of long-term heart rate variability biofeedback (HRVB) on psychophysiological responses, this study seeks to explore the short-term effects arising from a single HRVB session during and after paced breathing exercise. The research aligns with the neurovisceral integration model, emphasizing the link between heart rate variability (HRV) levels and cognitive performance. Therefore, a randomized controlled trial employing a between-subjects design was conducted with 38 participants. Each participant was assigned to either the paced breathing intervention group or the spontaneous breathing control group. The study assessed various parameters such as cardiac vagal tone, evaluated through vagally mediated HRV measures, and working memory, measured using the N-back task. Additionally, participants’ affective states were assessed through self-reported questionnaires, specifically targeting attentiveness, fatigue, and serenity. The results notably reveal enhancements in the working memory task and an elevated state of relaxation and attention following the HRVB session, as evidenced by higher averages of correct responses, serenity and attentiveness scores. However, the findings suggest that this observed improvement is not influenced by changes in cardiac vagal tone, as assessed using a simple mediation analysis. In conclusion, this study presents promising insights into the impact of a single HRVB session, laying the foundation for future research advancements in this domain.

**Supplementary Information:**

The online version contains supplementary material available at 10.1007/s10484-024-09624-7.

## Introduction

Within the intricate interplay of cardiac and cognitive domains, heart rate variability (HRV) emerges as a captivating physiological measure that offers valuable insights into the relationship between autonomic nervous system (ANS) and cognitive performance (Berntson et al., 1997; Thayer & Lane, 2000; Thayer et al., 2009). Reflecting the dynamic balance between sympathetic and parasympathetic influences on the heart, HRV encompasses the variation in time intervals between successive heartbeats (Berntson et al., 2009; Malik et al., 1996). This measure serves as an indicator of autonomic flexibility and regulatory capacity, with higher HRV suggesting a healthier and more adaptive ANS functioning (Ernst, 2017).

Six major domains of cognitive function have been postulated by the American Psychiatric Association: namely, complex attention, executive function, language, learning and memory, perceptual-motor function, and social cognition (Sachdev et al., 2014). Studies on executive function represent a growing field due to its direct association with activities of daily living (Chan et al., 2008; Starcke et al., 2016). Executive function refers to the higher cognitive skills underpinning self-control and goal-directed behaviour, including decision-making, problem-solving, and self-regulation. It comprises three primary areas: inhibitory control, working memory, and cognitive flexibility (Diamond, 2013). Inhibitory control involves the capacity to self-regulate one’s attention, actions, and emotions. Working memory involves the retention of information for a limited amount of time as it is being mentally processed. Cognitive flexibility refers to the mental ability to adapt to new situations or changes, and it is based on inhibitory control and working memory. From a neuroscientific perspective, the executive function processes are predominantly located in the prefrontal cortex of the frontal lobe and supported by connected brain structures (Blair, 2017).

Historically, the exploration of afferent cardiovascular activity towards the brain has been closely examined, particularly in relation to cognitive function (Porges, 1995). Notably, the seminal work by Lacey and Lacey (1970) established a link between heart rate deceleration and improved cognitive performance in sensory-intake tasks, as evidenced by faster reaction times (Lacey et al., 1974). Recognizing the complexity of the heat-brain connection, Wölk & Velden highlighted the significance of the pattern and stability of heart afferent input in influencing cognitive function on a micro-scale level, spanning 3–4 cardiac cycles (Wölk & Velden, 1987, 1989). More recently, this investigative approach has been expanded to explore the relationship between cardiovascular afferent activity and cognitive function on a macro-scale temporal pattern with longer time periods (McCraty et al., 2009; McCraty & Shaffer, 2015). Taking a theoretical approach, various models have emerged to elucidate the heart-brain interaction, revealing its profound impact on psychological responses and cognitive performance (Shaffer et al., 2014). Prominent examples include the polyvagal theory (Porges, 2011), the cardiac coherence model (McCraty & Childre, 2010), and the neurovisceral integration model (Thayer & Lane, 2000; Thayer et al., 2009). The latter model demonstrates the link between the prefrontal cortex and cardiac vagal tone, thus delineating the association between HRV and emotion, cognition, and mental well-being. An association between higher resting HRV measures and better emotion recognition was demonstrated by Quintana et al. (2012), indicating a relationship between the ANS and cognitive processes. One of the critical domains of the neurovisceral integration model is the relationship between cognitive function and HRV, particularly the root mean square of successive differences (RMSSD) and high frequency (HF) power (Thayer & Lane, 2000; Thayer et al., 2009). Forte et al. (2019) identified the major cognitive areas used in HRV studies by conducting a systematic review of the literature and concluded that, in general, higher resting HRV measures were associated with improved cognitive functioning, as examined in tasks assessing attention (Williams et al., 2016), global cognition (Frewen et al., 2013), and memory (Hansen et al., 2003, 2004).

While exploring ways to improve HRV, biofeedback through paced breathing exercises under slow respiratory rates emerged as a promising approach for enabling an individual to increase vagally-mediated HRV measures by activating the parasympathetic response (Lehrer et al., 2000; Khazan, 2013). The maximum HRV level, in which heart rate rhythms and breathing patterns are synchronised, is commonly referred to as the resonant frequency. Normal resonant frequency rates for adults range between 4.5 and 6.5 breaths/min (Lehrer, 2007). Further, heart rate variability biofeedback (HRVB) is an effective technique for building resilience and improving mental health and well-being in the long term (Lehrer & Gevirtz, 2014; Gevirtz, 2013; Laborde et al., 2019a). In a recent meta-analytic study, Lehrer et al. (2020) reported a significant and small-to-medium effect size regarding the efficacy of multiple-session HRVB in improving a wide variety of physical (e.g., asthma) and psychological symptoms (e.g., anxiety, depression). Moreover, preliminary evidence suggests that HRVB stimulates activity in the vagus nerve, which is a major parasympathetic nerve associated with relaxation and reduced stress levels (Gerritsen & Band, 2018). Initially, Lehrer et al. (2000) proposed a 10-session resonance breathing protocol to train individuals on HRVB techniques. Researchers have applied this protocol widely to investigate the long-term impact of HRVB on physical health, mental health, and cognitive performance. Nevertheless, Lehrer et al. (2013) simplified the training protocol and reduced the number of sessions to five for research and clinical purposes. Although it is advantageous to determine the resonant frequency for each individual, studies conducted in the past few years have indicated consistent physiological responses when breathing at a rate of 6 breaths/min (bpm; Van Diest et al., 2014; Zaccaro et al., 2018). However, further investigations are needed to understand the psychological responses with respect to the resonant frequency (see section “Cardiac Vagal Tone”).

To date, only a few studies have attempted to examine the short-term effects of a single HRVB session on the psychophysiological responses and cognitive performance (Laborde et al., 2019b, 2022b; Lin et al., 2020; Prinsloo et al., 2011, 2013; Schuman & Killian, 2019; Steffen et al., 2017; Wells et al., 2012; You et al., 2021a). Moreover, there is still controversy over the extent of HRVB influence on cognitive function, as discussed in a recent systematic literature review by Tinello et al. (2022). While there was a generally positive relationship between HRVB intervention and cognitive performance in the reviewed studies, there was a lack of evidence supporting that cognitive improvement can be directly attributed to an increase in HRV measures. This uncertainty stems from the fact that half of the studies examined (8 out of 16) did not present physiological data following the intervention. Therefore, the present study aimed to investigate the influence of HRVB on a range of affective states (attentiveness, fatigue, and serenity), executive function (cognitive performance in a working memory task), and cardiac vagal tone as a physiological measure during and after the intervention. In assessing cardiac vagal tone, RMSSD was selected as the primary measure due to its reduced sensitivity to respiration compared to HF power (Shaffer & Ginsberg, 2017). The hypotheses for this study are as follows, where the HRVB intervention was conducted as a short-term single paced-breathing session:

*H1*: HRVB improves the affective states associated with attentiveness, fatigue, and serenity during and after the paced-breathing session.
*H2*: HRVB improves the working memory after the paced-breathing session.
*H3*: HRVB improves cardiac vagal tone during and after the HRVB activity.
*H4*: The improvement of cognitive performance after the HRVB activity is mediated by changes in the cardiac vagal tone.

## Materials and Methods

### Participants

The study aimed to recruit a total of 34 participants based on a priori power analysis for a repeated measures ANOVA with a between-subjects factor design using G*power. The analysis considered a statistical power ($1-\beta$) of 80%, a significance level ($\alpha$) of .05, a correlation among repeated measures of .50, and the ability to detect a large effect size (*f*) of .40.

In total, 44 healthy adults aged 23–62 years were recruited to participate in the study. These individuals were randomly assigned to either the intervention or control group. Recruitment efforts targeted individuals from Hamad Bin Khalifa University (HBKU) in Qatar, as well as the general community through email advertisements and personal invitations. To ensure the validity of the findings, exclusion criteria were applied, which included physical health conditions associated with cardiovascular or respiratory diseases, diagnosed psychiatric conditions, and an age outside the range of 18–65 years at the time of recruitment. Additionally, participants were instructed to avoid consuming caffeine, smoking, and eating heavy meals for 2 h prior to the study. They were also advised against engaging in intense physical workouts for 24 h to minimize any potential confounding effects on the physiological responses (Laborde et al., 2017; Quintana et al., 2016). The study protocol was approved by the Institutional Review Board at Qatar Biomedical Research Institute at HBKU (QBRI-IRB-2021-03-088). Before participating in the study, all participants were provided with detailed information about the nature of the experiment and provided their informed consent by signing the form digitally. Following data collection, the HRV recordings were visually inspected and filtered to remove noise and artifacts. As a result, data from six participants were excluded due to poor signal quality, where the noise level exceeded 5% of the recording. The remaining dataset consisted of 38 participants, of which 20 were women (mean age: 35.5 ± 11) and 18 were men (mean age: 34.4 ± 9.86).

### Experiment Design

A randomized controlled trial was conducted to investigate the impact of HRVB through paced breathing on various affective states, executive function, and physiological measures in healthy individuals. The study employed a mixed-factorial design with two independent variables: group (between-subjects) and time (within-subjects). Participants were randomly assigned to one of two groups: (1) the HRVB group, which received paced breathing as a biofeedback intervention, and (2) the control group, which engaged in normal breathing without any intervention.

**Fig. 1:**
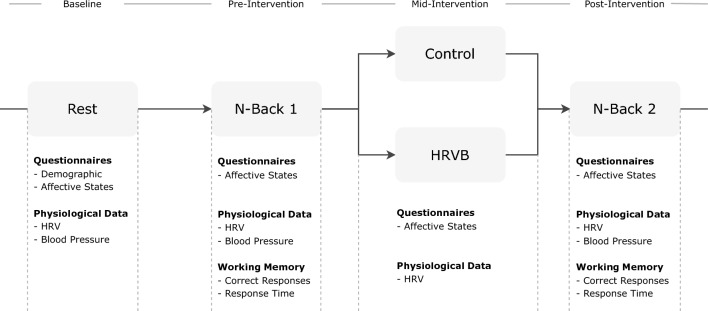
A flowchart for the experimental protocol

Data collection included HRV measurements and affective state questionnaires at four time points throughout the study: (1) baseline, (2) pre-intervention (during the first cognitive task), (3) mid-intervention, and (4) post-intervention (during the second cognitive task). HRV data were collected using a photoplethysmography (PPG)-based sensor attached to the non-dominant hand, while blood pressure was measured using an upper arm cuff device. Figure 1 illustrates the experimental protocol.

#### Questionnaires

All participants filled out the HRV-related questionnaire developed by Quintana et al. (2016) as a screening survey to assess their eligibility. Furthermore, participants completed a set of questionnaires during baseline, including questions related to demographics; Positive and Negative Affect Schedule (PANAS) to assess emotional state (Watson & Clark, 1994); Depression, Anxiety, and Stress Scale (DASS-21) to assess depression, anxiety, and stress (Lovibond & Lovibond, 1995); International Physical Activity Questionnaire-Short Form (IPAQ-SF) to measure physical activity (Craig et al., 2003); and Pittsburgh Sleep Quality Index (PSQI) to assess sleep quality (Buysse et al., 1989).

In addition, affective states were measured during the experiment via self-reported questionnaires on attentiveness, fatigue, and serenity using the PANAS-Expanded Form (PANAS-X), which is based on a multiple-item Likert scale. Each component of the PANAS-X consisted of multiple terms measuring affective states at that moment, and these terms had to be rated 1 for “not at all” to 5 for “extremely”. All questionnaires used were presented in the English and Arabic languages as most of the participants were non-native English speakers. The Arabic versions of the questionnaires were obtained as follows: PANAS (Davis et al., 2020), DASS (Ali et al., 2017), PSQI (Suleiman et al., 2010), and IPAQ (Helou et al., 2017). The remaining demographic and HRV-related questionnaires were translated by the first author.

#### Working Memory

Executive function was assessed using the N-back task, a computer-based cognitive test for evaluating the working memory capacity (Kirchner, 1958). It involved presenting a series of random alphabetical letters on a laptop screen. Participants were required to determine if the current letter matched the one presented in the previous N trials. The N-back task was implemented using the PsyToolkit web-based platform, with N set to 2 (Stoet, 2010, 2017). Each letter was displayed for 1800 ms, followed by a 500-ms blank screen period. Participants were instructed to press the “M” key on the keyboard if there was a match, and no response was required otherwise.

The cognitive task yielded three possible responses: correct responses, missed responses, and false alarms. Additionally, the reaction time was measured as the duration it took for participants to correctly press the “M” key after the letter appearance, indicating a match response. The N-back task consisted of two blocks, each comprising 50 trials. Randomization of letters was performed to eliminate potential biases. Participants underwent a training session with 25 trials to familiarize themselves with the task before completing it. To assess cognitive performance improvement, participants completed the N-back task at two time-points: pre-intervention and post-intervention. Hence, correct responses and reaction time were used to evaluate working memory capacity performance.

#### Physiological Data

HRV was recorded using the CorSense device by Elite HRV^1^, which could be conveniently attached to the finger and had a sampling rate of 500 Hz. Prior to each measurement, a 20-s stabilization period was allowed to ensure the heart rate had leveled out. In the case of any technical issues, HRV was recorded for 6 min to ensure a minimum recording length of 5 min. Participants were instructed to minimize hand movements to maintain a high-quality signal. For the HRV analysis, the Systole Python package was used to compute the time-domain and frequency-domain measures (Legrand & Allen, 2022). All signals were visually inspected and filtered using the adaptive threshold artefact detection and moving window average correction methods (Lipponen & Tarvainen, 2019). Moreover, blood pressure was measured for all participants following each cognitive task using an OMRON M7 Intelli IT^2^ cuff wrapped around the upper arm.

#### Procedure

**Fig. 2:**
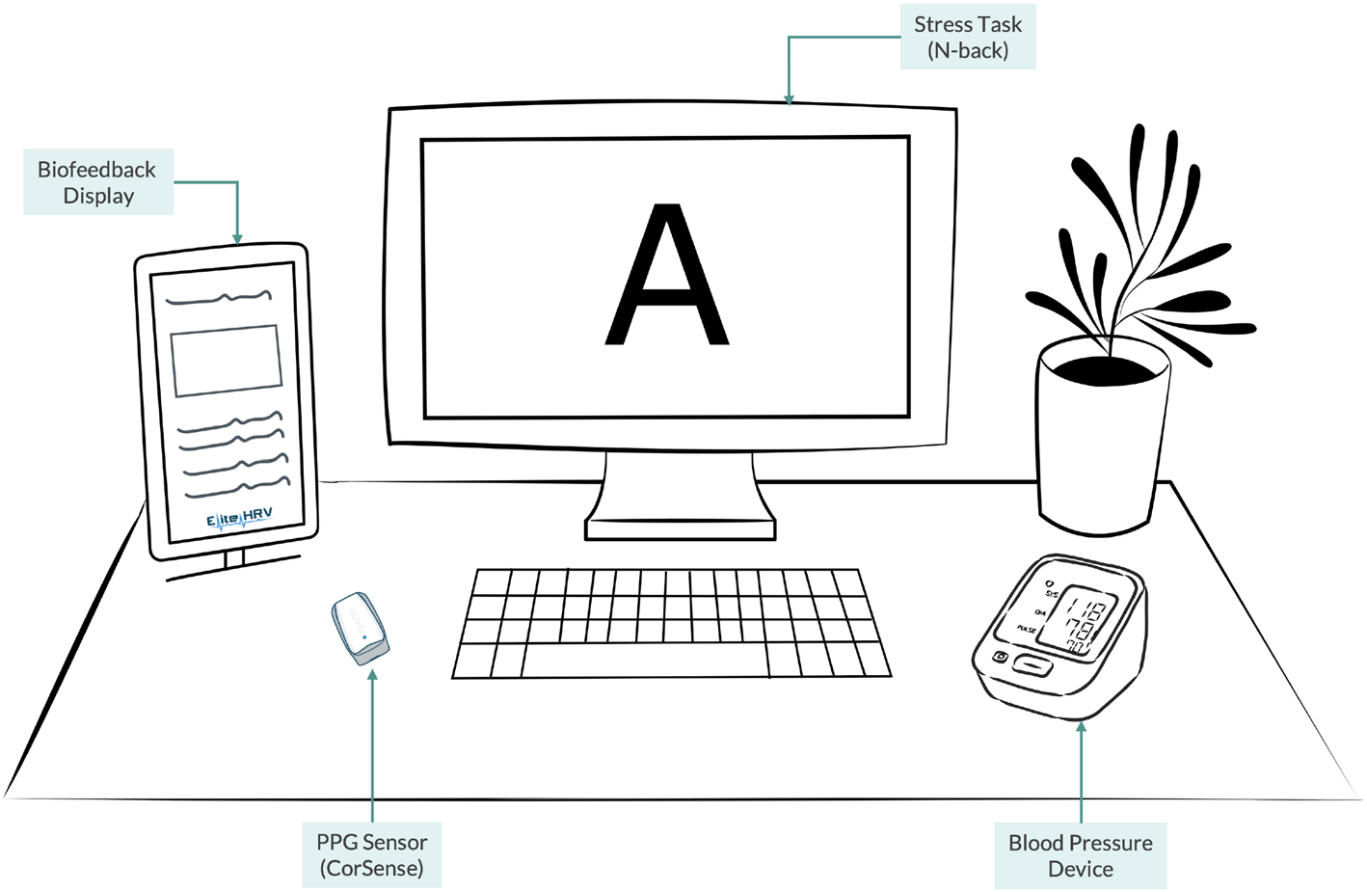
The experiment setup includes the CorSense sensor, blood pressure device, biofeedback interface, and cognitive task screen

The experimental sessions were conducted during the daytime (9:00 am to 2:00 pm) in the human-computer interaction (HCI) lab at HBKU, with each session lasting 45 min. The HCI lab is a quiet small room specifically designed for in-person experimental studies. A schematic diagram of the overall experimental setup is shown in Fig. 2. The experiment protocol involved collecting psychophysiological data at four time points: (1) baseline, (2) pre-intervention, (3) mid-intervention, and (4) post-intervention.

Upon arrival at the lab, participants were requested to sign the consent form and complete baseline questionnaires concerning demographic information, anxiety, depression, stress, emotional state, physical fitness, and sleep quality. Subsequently, a 6-min HRV recording was taken while the participants were at rest, followed by a blood pressure measurement. After the baseline measurements, participants began the first cognitive task presented on a laptop screen, with HRV data also recorded during this phase. Participants were left alone in the room to complete the cognitive tasks, while the researcher remained present between phases to address any concerns and ensure there were no technical issues. Following that, participants received a randomly generated message on the screen indicating their group assignment to either Group 1 or Group 2 (i.e., control or HRVB, respectively).

In the HRVB group, participants engaged in a paced breathing exercise for 6 min by following a breathing guide displayed on an iPad screen using the Elite HRV deep breathing feature. The visualization of the sinusoidal wave of the HRV signal during breathing, along with instructional audio guiding inhalation and exhalation, provided the biofeedback element to the participants. A brief explanation of the relationship between HRV and breathing was provided to make participants aware of any deviations from the expected sinusoidal pattern. Participants were instructed that, visually, HRV should exhibit an increase during inhalation and a decrease during exhalation, forming a wave-like pattern. Prior to the paced breathing activity, a 2-min training session was conducted to ensure participants could correctly perform the breathing exercise. The paced breathing activity utilized a prolonged exhalation period with a ratio of 4 s of inhalation and 6 s of exhalation (6 bpm; Khazan, 2013). Longer exhalations have been shown to be a stimulating protocol for notable improvements in cardiac vagal tone (Van Diest et al., 2014). Participants in the control group were instructed to sit quietly for 6 min and breathe normally, similar to the baseline phase. Finally, all participants completed the second cognitive task and proceeded to a debriefing session.

#### Analysis Plan

In this experimental study, we analyzed the statistical mean differences between groups to investigate the impact of the intervention on affective states (H1) using a two-way mixed analysis of variance (ANOVA), followed by planned contrasts. The independent variables were defined by the group factor (HRVB vs. control; between-subjects) and the time factor (pre-, mid-, post-intervention; within-subjects), while the dependent variables included scores for attentiveness, fatigue, and serenity. To assess the effects of the intervention on cognitive performance (H2), we employed independent sample t-tests to compare the difference between post and pre scores of the correct responses and reaction time between both groups. Additionally, we analyzed the statistical mean differences between groups to investigate the impact of the intervention on RMSSD (H3) using two-way mixed ANOVAs, followed by planned contrasts for post-hoc analysis. The statistical analyses for H1–H3 were conducted using JASP software (JASP Team, 2023). Finally, a simple mediation model analysis was performed using the PROCESS package in R, developed by Hayes (2022) to examine the mediating role of cardiac vagal tone in the association between the HRVB intervention and the improvement in cognitive performance (H4).

As a measure of effect size, the omega squared ($\omega ^2$) was reported for the ANOVA, with values of .01, .06, and .14, indicating small, medium, and large effect sizes, respectively. Moreover, Hedges’ g was reported for all pairwise comparisons, with the values of .20, .50, and .80, indicating small, medium, and large effect sizes, respectively (Cohen, 1988). The confidence interval (95% CI) was reported in the statistical analyses, as appropriate.

## Results

### Descriptive Statistics

A descriptive statistical overview of demographic characteristics and baseline psychophysiological measures based on the group are shown in Supplementary Table A1. At baseline, there were no significant differences between the groups in terms of age; blood pressure; body mass index; depression, anxiety, and stress (DASS-21); experience with deep breathing; experience with meditation; HRV measures; physical activity level (PSQI); positive and negative affective states (PANAS-X); or sleep quality (PSQI; see Table A1). Gender was balanced, with 10 women and nine men in each group. The effect of gender on the baseline HRV measures was also examined, and the independent t-test revealed no significant mean differences (*p*-values > .32 for all).

At baseline, the correlations among variables presented in Supplementary Table A1 were calculated using the Pearson’s correlation coefficient. There were significant positive relationships between the negative affective states measured by the PANAS and DASS-21 (depression: r = .69, anxiety: r = .72, stress: r = .71, all  *p* < .05) and sleep quality measured by the PSQI (r = .39, *p* < .05). There was a significant inverse relationship between age and the baseline SDNN measure. However, there were no significant relationships between age and the remaining HRV measures. Additionally, the correlation coefficients between RMSSD and self-reported affective states at baseline were found to be non significant (attentiveness: r = .08, fatigue: r = − .19, serenity: r = .22, all *p* > .05; refer to Supplementary Table A2 for additional HRV metrics).

### Affective States

The first hypothesis posed that a single session of HRVB would have positive effects on levels of perceived attentiveness, fatigue, and serenity. A two-way mixed ANOVA was performed to assess the impact of the group (HRVB vs. control; between-subjects) and time (pre-, mid- and post-intervention; within-subjects) on the affective states reported from the PANAS-X questionnaire. The Greenhouse–Geisser correction was applied when the sphericity assumption was violated. All scores at the pre-, mid-, and post-intervention time points were normalised by subtracting the scores reported at baseline. Shapiro–Wilk test was conducted to ensure normality (*p* > .05 for all) and homogeneity of variances was found for all PANAS-X components, as assessed by Levene’s test (*p* > .36 for all).

*Attentiveness*: There was a statistically significant interaction between group and time on the attentiveness scores, F(2, 72) = 3.6, *p* = .032, $\omega ^2$ = .014. Planned contrasts revealed that attentiveness score was significantly greater in the HRVB group compared to the control group at mid-intervention (M_diff_ = 1.79, SE = 0.73, *p* = .018, Hedges’ g = 0.81) and post-intervention (M_diff_ = 2, SE = 0.73, *p* = .008, Hedges’ g = 0.91).
*Fatigue*: There was no statistically significant interaction between group and time on the fatigue scores, F(2, 72) = 1.02, *p* = .37, $\omega ^2$=.00. The main effect of time showed a statistically significant difference in mean fatigue score at the different time points, F(2, 72) = 7.38, p = .001, $\omega ^2$ = .035. Planned contrasts revealed a significant increase in the fatigue score of the HRVB group at mid-intervention compared to the pre-intervention (M_diff_ = 1.95, SE = .56, *p* <.05, Hedges’ g = 0.53).
*Serenity*: There was a statistically significant interaction between group and time on the serenity scores, F(1.48, 53.1) = 15.6, *p* <.001, $\omega ^2$ = .08. Planned contrasts revealed that serenity score was significantly greater in the HRVB group compared to the control group at mid-intervention (M_diff_ = 3.84, SE = 0.82, *p* < .001, Hedges’ g = 1.7) and post-intervention (M_diff_ = 3.42, SE = 0.82, *p* = .002, Hedges’ g = 1.4).

### Working Memory

**Fig. 3:**
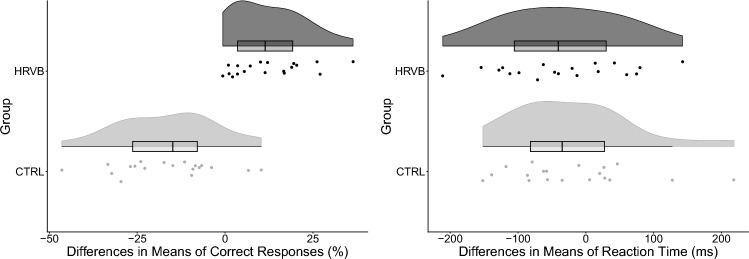
Results of the difference in cognitive performance (post–pre)

The second hypothesis stated that a single session of HRVB would improve the cognitive performance of the working memory task. An independent-samples t-test was performed to assess the differences in correct responses and reaction time between control and HRVB. The correct responses and reaction time were computed as the change in scores between post-intervention and pre-intervention. The Shapiro–Wilk test was conducted to ensure normality on the correct responses and reaction time for each group. The results were non-significant, indicating that both metrics were approximately normally distributed (*p* > .11 for all). The variance was homogeneous between the groups for both metrics, as assessed by Levene’s test (*p* > .56 for all). The results revealed that the correct responses score was higher in the HRVB group compared to the control group, a statistically significant difference of 29.2% (95% CI 20.8–37%), t(36) = 7.2, *p* < .001, Hedges’ g = 2.2. In contrast, there was no significant difference between both groups in reaction time t(36) = .49, *p* = .63, Hedges’ g = .16. Figure 3 shows raincloud plots for the differences in correct responses and reaction time (Allen et al., 2021).

### Cardiac Vagal Tone

The third hypothesis stated that a single HRVB session would improve the cardiac vagal tone as indexed by the RMSSD measure. A two-way mixed ANOVA was performed to assess the impact of the group (HRVB vs. control; between-subjects) and time (pre-, mid- and post-intervention; within-subjects) on the RMSSD while considering age and baseline measurements as covariates. Following ANOVA computation, planned contrasts was carried out to discern any significant pairwise differences. To ensure normality, the Shapiro–Wilk test was calculated for the log-transformed HRV values of each experimental condition. The results across all conditions were non-significant, indicating that the RMSSD measures were approximately normally distributed (*p* > .05 for all). Mauchly’s test of sphericity indicated that the assumption of sphericity was met for the two-way interaction, ($\chi ^2(2)=.29$, *p* = .86). Moreover, we conducted an exploratory and supplementary analysis to examine the impact of the biofeedback session on additional HRV measures including MeanRR, SDNN, and HF power, using two-way mixed ANOVAs. The mean differences of the HRV measures are presented in their non-transformed form (i.e., absolute values) for simplicity and ease of interpretation, while the statistical analyses were performed on the log-transformed data. To assess participants’ adherence to the instructed slow-breathing protocol (6 bpm), the respiration rate (RESP) was estimated using Kubios HRV standard software based on the observed peak frequency in the spectral analysis of the HRV signal (Bailón et al., 2007; Shaffer & Meehan, 2020). Table 1 shows the descriptive statistics for all examined HRV measures and the estimated respiration rate.

**Table 1:** Descriptive statistics for the physiological measures

	Pre		Mid		Post	
Measure	HRVB	Control	HRVB	Control	HRVB	Control
*RMSSD*						
ms	54.4 (43.1)	48.1 (18.9)	58.5 (40.5)	44.6 (31.1)	69.6 (64.6)	53.5 (35.8)
log	3.73 (0.75)	3.8 (0.38)	3.86 (0.66)	3.6 (0.64)	3.89 (0.85)	3.79 (0.63)
*MeanRR*						
ms	743.1 (105.6)	723.3 (77)	748.53 (104)	738.2 (79.8)	778.7 (104)	731.4 (78.6)
log	6.60 (0.15)	6.58 (0.1)	6.66 (0.13)	6.6 (0.1)	6.65 (0.13)	6.59 (0.11)
*SDNN*						
ms	66.9 (47.4)	51.7 (25)	106 (47.9)	52.8 (21.2)	79.2 (64)	56 (34)
log	3.99 (0.66)	3.86 (0.42)	4.56 (0.49)	3.90 (0.39)	4.07 (0.8)	3.88 (0.54)
*HF power*						
ms	1755 (2763)	1187 (2201)	1674 (2048)	1120 (1185)	3323 (5286)	1256 (1934)
log	6.28 (1.67)	6.15 (1.41)	6.54 (1.55)	6.54 (1.09)	6.62 (1.99)	6.19 (1.5)
*RESP*						
bpm	9.45 (1.26)	9.98 (1.25)	6.08 (0.28)	9.54 (1.12)	8.54 (1.10)	9.53 (1.2)

*Note* Data is represented by the format of Mean (SD)

*RMSSD*: There was no statistically significant interaction between group and time on the RMSSD measure, F(2, 68) = 1.42, *p* = .25, $\omega ^2=.006$. Likewise, there were no significant main effects of either time F(2,68) = 0.29, *p* = .75 $\omega ^2=.00$ or group F(1, 34) = 4.15, *p* = .05, $\omega ^2=.04$. The mean differences in RMSSD between the HRVB and control groups were observed at mid-intervention (Adjusted M_diff_ = 22.95 ms, SE = 12.17 ms, Hedges’ g = .38), and post-intervention (Adjusted M_diff_ = 19.81 ms, SE = 12.17 ms, Hedges’ g = .23).
*MeanRR*: There was no statistically significant interaction between group and time on the MeanRR measure, F(2, 68) = 2.25, *p* = .11, $\omega ^2=.013$. Similarly, there were no significant main effect of group, F(1,34) = 1.08, *p* = .31, $\omega ^2=.00$. However, there was a significant main effect of time, F(2, 68) = 8.65, *p* <.001 $\omega ^2=.08$. Planned contrasts revealed than MeanRR for the HRVB group was greater at mid-intervention (Adjusted M_diff_ = 40.2 ms, SE = 10.7 ms, t(68) = 3.76, *p* <.001) and post-intervention (Adjusted M_diff_ = 34.82, SE = 10.7, t(68) = 3.25, *p* = .002) compared to pre-intervention.
*SDNN*: There was a statistically significant interaction between group and time on the SDNN measure, F(2,68) = 4.21, *p* = .019, $\omega ^2=.038$. Planned contrasts revealed that SDNN at mid-intervention was greater in the HRVB group compared to the control group (Adjusted M_diff_=.695, SE = .173, t = 4.01, *p* = .002). Furthermore, SDNN for the HRVB group was significantly elevated at the mid-intervention compared to both pre- and post-intervention (*p* <.001).
*HF Power*: There was no statistically significant interaction between group and time on the HF power measure, F(2, 68) = 0.24, *p* = .79, $\omega ^2=.00$. Similarly, there were no significant main effects of either time, F(2, 68) = 0.12, *p* = .89 $\omega ^2=.00$, or group F(1, 34) = 0.66, *p* = .42, $\omega ^2=.00$.

The final hypothesis (H4) suggested that the improvement in cognitive performance following the HRVB session is mediated by the cardiac vagal tone. A simple mediation model analysis was performed using the bootsrapping method of the 95% CI with a bootstrapping sample of 10,000. The outcome variable for the analysis was the correct responses, the predictor variable was the HRVB intervention as represented by the group assignment (i.e., control coded as 0 and HRVB coded as 1), and the mediator variable was the RMSSD measure. The findings indicate a statistically significant total effect (c = 8, *p* = .01, 95% CI [1.62, 14.38]), and similarly, the direct effect is also statistically significant (c = 7.7, *p* = .02, 95% CI [1.09, 14.47]). Notably, the indirect effect of the HRVB intervention on cognitive performance was not statistically significant (95% CI [$-$1.33, 1.73]).

## Discussion

### Affective States

Several self-reported affective states were investigated in this study, including attentiveness, fatigue, and serenity. The findings partially support H1 because only attentiveness and serenity components of the PANAS-X questionnaire revealed positive results for the levels in attention and relaxation during and after the HRVB intervention. These results are in line with those reported in previous studies focused on attention control (de Bruin et al., 2016) and relaxation (Clamor et al., 2016; Lin et al., 2020; Prinsloo et al., 2013; Van Diest et al., 2014; Zaccaro et al., 2018). The present study extended these findings by examining participants’ subjective perception of their own attentiveness and relaxation following the cognitive task and HRVB session. Regarding serenity levels, Lehrer and Gevirtz (2014) stipulated that the mechanisms underlying HRVB induce a relaxation response by stimulating parasympathetic activity mediated by vagal tone. The attentiveness score outcomes in this study suggest a link to improved performance in the cognitive task after the biofeedback intervention. During the debriefing session, one participant in the HRVB group commented: “The deep breathing practice helped me think clearly about strategies to solve the cognitive task”.

Although there was no statistically significant interaction observed in terms of the impact of paced breathing on perceived fatigue between the two groups, the HRVB group reported a significant higher average score immediately after the intervention compared to pre-intervention representing a medium effect size. This finding could be due to the participants’ lack of familiarity with paced breathing exercises, which resulted in a dyspnoeic or uncomfortable experience. In the same vein, You et al. (2021b) noted an elevation in perceived stress following a series of three 5-min paced breathing exercises in their study. This increase in stress levels was attributed to the discomfort experienced during paced breathing, which is a common occurrence among individuals unfamiliar with the practice.

### Working Memory

For the cognitive performance aspect of this study, the findings partially support H2 because the HRVB group performed better in the cognitive task compared to the control group, as assessed by the correct responses. However, no differences were found with respect to reaction time. More specifically, the HRVB group performed better than the control in the second N-back task, which assessed participants’ working memory capacity. These results are consistent with Prinsloo et al.’s (2011) findings regarding improvement in cognitive performance (i.e., inhibitory control measured using a Stroop task) after a single HRVB session. This observed significant increase in correct responses could be theoretically attributed to the HRVB intervention, which stimulated the vagus nerve. In particular, previous studies have linked the activation of parasympathetic activity with improvement in the working memory and attention-based tasks (Forte et al., 2019; Hansen et al., 2003, 2004). However, there was no significant difference in reaction time between the two groups post-intervention in the present study, which is in direct contrast to Prinsloo et al. (2011). This rather contradictory result may be due to the experimental protocol as the previous study advised participants to consider speed when responding, whereas participants were not similarly advised in this study. Another possible explanation may be that this study looked at reaction time for correct responses to accurately quantify processing speed (Ratcliff, 1993). Mahinrad et al. (2016) found that poor processing speed and long reaction time in cognitive functioning evaluated by a Stroop task were associated with low HRV measures. However, the authors analysed HRV signals using a 10-s segment, while the present study analysed HRV signals using a 5-min segment. There is a well-established trade-off between accuracy and reaction time in cognitive activities: individuals compromise accuracy for speed, or vice versa (Donkin et al., 2014; Franzon & Hugdahl, 1987; Wylie et al., 2009). Further, Mahinrad et al. (2016) focused on a specific age group (i.e., older participants), thus limiting the generalisability of their findings to younger age groups.

### Cardiac Vagal Tone

At a physiological level, the findings do not support H3 due to insufficient evidence indicating improvements in cardiac vagal tone during and after the HRVB intervention. Although statistical analysis did not show a significant difference in vagal tone, measured by RMSSD, between the two groups at various time points, the HRVB group exhibited higher RMSSD values compared to the control group at mid-intervention and post-intervention, with small effect sizes. However, there was no significant association between vagal tone and improved working memory, as evidenced by the non-significant results of the mediation analysis, thereby not supporting H4. These findings are in agreement with prior research demonstrating that a single-paced breathing session does not sufficiently improve RMSSD after the session (Laborde et al., 2019b; You et al., 2021a) and the improvement in cognitive performance is not mediated by RMSSD (Laborde et al., 2022a). Consequently, the present study obtained a similar conclusion regarding post-intervention RMSSD and vagal tone, despite the previous studies not including a biofeedback component in their design. However, exploring variations in the patterns and amplitude of cardiovascular vagal afferent input during the paced breathing activity could provide valuable insights into the mechanisms underlying the study outcomes (McCraty & Childre, 2010). This can potentially offer a more nuanced understanding of how HRVB influenced cognitive performance and changes in affective states.

Contrary to the results reported in You et al. (2021a), the present study did not find significant group differences in RMSSD during the intervention; however, these results are in line with those of Laborde et al. (2019b). While this study implemented a paced breathing rate similar to that in You et al. (2021a), the discrepancy in results could be due to the number and duration of paced breathing exercises (e.g., three 5-min sessions), type of participant (e.g., athletes), or type of control (e.g., watching television).

### Limitations

There are a number of limitations in the present study related to the biofeedback protocol. First, the lack of participants’ familiarity with paced breathing exercises may have posed challenges in correctly performing the activity. Although the exercise duration was intentionally selected to be short (6 min) to minimise discomfort in participants unfamiliar with the exercise, a better strategy may be to adopt multiple consecutive short sessions with breaks in between, as in Laborde et al. (2022b) and You et al. (2021a). Furthermore, real-time respiratory monitoring using a respirometer during the intervention could have provided stronger evidence of participant compliance to the instructed slow-breathing protocol (6 bpm), potentially boosting the interpretability of the study’s findings (Shaffer & Meehan, 2020). Second, all participants performed the breathing exercise at the same rate of 6 breaths/min rather than determining the resonant frequency for each participant. Although several studies have indicated similar physiological behaviour with 6 breaths/min during the exercise, Steffen et al. (2017) observed differences in self-reported mood between breathing at resonant frequency and one breath per minute higher than the determined resonant frequency. Consequently, future studies could investigate the distinctions between resonant frequency and breathing at a fixed rate after the paced breathing exercise at a psychological level.

## Conclusions

In summary, this study explores the impact of HRVB on psychophysiological measures, specifically focusing on self-reported affective states, cardiac vagal tone, and cognitive performance related to working memory. Despite the absence of an associated increase in vagal tone, as indicated by RMSSD, cognitive performance displayed promising improvement following the biofeedback intervention, evident in correct responses and attentiveness scores. The lack of increase in vagal tone could be explained by several factors, such as the biofeedback protocol, duration of the biofeedback session, or participants’ lack of familiarity with paced breathing. An additional prominent finding is the improvement in relaxation levels measured via self-reported serenity scores after the biofeedback intervention. For a more comprehensive understanding of vagal tone within the neurovisceral integration model, future studies could incorporate alternative cognitive stress tasks that impose a higher mental workload, such as the dual N-back task involving auditory and visual stimuli. With respect to the effects of paced breathing on vagal tone, the biofeedback protocol can be similarly improved by determining the resonant frequency for each participant or incorporating a longer paced breathing session﻿.

### Supplementary Information

Below is the link to the electronic supplementary material.

Supplementary file 1 (pdf 77 KB)

## Data Availability

The data that support the findings of this study are available from the corresponding author upon reasonable request.

## Abbreviations

RMSSD: Root mean square of successive differences
HRV: Heart rate variability
HRVB: Heart rate variability biofeedback
HF: High frequency
ANS: Autonomic nervous system
DASS: Depression anxiety stress scale
PANAS: Positive and negative affect schedule
PSQI: Pittsburgh sleep quality index
IPAQ: International physical activity questionnaire
SDNN: Standard deviation of all normal to normal (R‐R) intervals

## Footnotes

1 https://elitehrv.com/corsense.

2 https://omron-healthcare.com/products/m7-intelli-it/.
