# Supplementary material for: Short-Term Effects of Heart Rate Variability Biofeedback on Working Memory
Source: Appl Psychophysiol Biofeedback. 2024 Feb 16;49(2):219–31. doi: 10.1007/s10484-024-09624-7 (PMC11101506; doi:10.1007/s10484-024-09624-7)
Supplement: Supplementary file 1 — (pdf 77 KB) [file 10484_2024_9624_MOESM1_ESM.pdf]

Appendix A Supplementary Data

Table A1 Demographic and Baseline Characteristics by Group (N=38)

	**Total**<br>mean (SD)	**Control**<br>mean (SD)	**HRVB**<br>mean (SD)	**t**	**p**
Age	34.9 (10.3)	33.6 (6)	36.3 (13.4)	-0.78	.44
Body Mass Index	25.7 (4.7)	25.6 (5.1)	25.8 (4.3)	-0.13	.90
**PANAS**					
Positive Affect	24.4 (7.9)	26.2 (6.6)	22.5 (8.9)	1.45	.16
Negative Affect	8.95 (6.5)	8.7 (5.9)	9.2 (7.2)	-0.20	.84
**DASS-21**					
Depression	1.58 (1.5)	1.63 (1.5)	1.53 (1.5)	0.22	.83
Anxiety	2.2 (1.6)	2.2 (1.5)	2.1 (1.5)	0.21	.84
Stress	1.1 (1.4)	1.1 (1.4)	1.1 (1.4)	0.00	1.0
**PSQI**					
Sleep Quality	6.66 (3.4)	7.21 (3.7)	6.11 (3.1)	1.0	.32
**IPAQ**					
MET	1547 (2196)	1092.3 (1152)	2002 (2855.3)	-1.29	.21
kCal	1848 (2765)	1198.8 (1068.3)	2497.3 (3699.5)	-1.47	.16
**HRV Time**					
MeanRR	783 (104)	766 (84)	800 (121)	-1.0	.32
RMSSD	46 (49)	52 (44)	40 (33)	0.97	.34
SDNN	60.4 (33)	61 (36)	59 (31)	0.18	.86
**HRV Frequency**					
LF Power	1557 (2799)	1949 (3277)	1165 (2246)	0.86	.40
HF Power	1266 (1707)	1368 (1856)	1164 (1588)	0.36	.72
LF/HF	1.8 (1.6)	2.1 (1.9)	1.6 (1.3)	0.97	.34
Total Power	3691 (4992)	4308 (5820)	3075 (4068)	0.76	.45

Table A2 Pearson Correlation Coefficients for HRV Measures and Affective States at Baseline

	**Attentiveness**		**Fatigue**		**Serenity**	
	**r**	**P**	**r**	**P**	**r**	**P**
**RMSSD**	.08	.61	-.19	.24	.22	.19
**MeanRR**	-.13	.42	.30	.06	.44	.01
**SDNN**	.16	.35	-.13	.43	.21	.20
**HF Power**	.08	.64	-.11	.53	.15	.36
